# Development of Resistance towards Artesunate in MDA-MB-231 Human Breast Cancer Cells

**DOI:** 10.1371/journal.pone.0020550

**Published:** 2011-05-26

**Authors:** Beatrice Bachmeier, Iduna Fichtner, Peter H. Killian, Emanuel Kronski, Ulrich Pfeffer, Thomas Efferth

**Affiliations:** 1 Department of Clinical Chemistry and Clinical Biochemistry, Ludwig-Maximilians-University, Munich, Germany; 2 Functional Genomics, Advanced Biotechnology Center, Genoa, Italy; 3 Department of Experimental Pharmacology, Max Delbrück-Center for Molecular Medicine, Berlin, Germany; 4 Department of Pharmaceutical Biology, Institute of Pharmacy and Biochemistry, Johannes Gutenberg University, Mainz, Germany; University of Illinois at Chicago, United States of America

## Abstract

Breast cancer is the most common cancer and the second leading cause of cancer death in industrialized countries. Systemic treatment of breast cancer is effective at the beginning of therapy. However, after a variable period of time, progression occurs due to therapy resistance. Artesunate, clinically used as anti-malarial agent, has recently revealed remarkable anti-tumor activity offering a role as novel candidate for cancer chemotherapy. We analyzed the anti-tumor effects of artesunate in metastasizing breast carcinoma *in vitro* and *in vivo*. Unlike as expected, artesunate induced resistance in highly metastatic human breast cancer cells MDA-MB-231. Likewise acquired resistance led to abolishment of apoptosis and cytotoxicity in pre-treated MDA-MB-231 cells. In contrast, artesunate was more cytotoxic towards the less tumorigenic MDA-MB-468 cells without showing resistance. Unraveling the underlying molecular mechanisms, we found that resistance was induced due to activation of the tumor progression related transcription factors NFκB and AP-1. Thereby transcription, expression and activity of the matrix-degrading enzyme MMP-1, whose function is correlated with increased invasion and metastasis, was up-regulated upon acquisition of resistance. Additionally, activation of the apoptosis-related factor NFκB lead to increased expression of ant-apoptotic *bcl2* and reduced expression of pro-apoptotic *bax*. Application of artesunate *in vivo* in a model of xenografted breast cancer showed, that tumors growth was not efficiently abolished as compared to the control drug doxorubicin. Taken together our *in vitro* and *in vivo* results correlate well showing for the first time that artesunate induces resistance in highly metastatic breast tumors.

## Introduction

Breast cancer belongs to the most fatal cancer types in industrialized countries [1]. While treatment options have considerably improved over the past decades, cure from the disease is still not a reality for all women suffering from breast cancer. Among the reasons for this situation are the development of drug resistance and severe side effects of chemotherapy, which still are unresolved problems in clinical oncology. Therefore, the search for novel anti-cancer compounds with improved features is mandatory.

A couple of years ago, we focused our efforts on artesunate [2], [3]. This is a semi-synthetic derivative of artemisinin, the active principle of *Artemisia annua* L. Artemisinin and its derivatives are valuable drugs treating multidrug-resistant *Plasmodium falciparum* and *P. vivax* infections. In addition to their efficacy in malaria treatment, they are cytotoxic towards cancer cells and multidrug-resistant tumor cells. More than 70 cell lines from different tumor types have been reported to be inhibited by artesunate and its related compound artemisinin [2], [4]. Over-expressing ATP-binding cassette-type drug transporters (MDR1/P-gp, MRP1, BCRP) do not reveal cross-resistance to artesunate [4]. We have also shown that normal cells are minimally or not affected by artesunate [5]. In addition, there are several reports by us and others that artesunate and artemisinin inhibit tumor growth in xenograft tumors *in vivo* [6], [7], [8], [9]. Case reports on the activity of this drug class in tumor patients [10] and a clinical study on 120 non-small cell lung cancer prove the anticancer activity of artesunate [11].

Despite the far-reaching lack of resistance in malaria and cancer, the first reports appeared concerning development of resistance in *Plasmodia* [12], [13], [14] implying that resistance to artesunate may also occur in cancer cells. To address the question of development of artesunate resistance in cancer cells, we have chosen breast cancer as suitable tumor type. The response rates of breast cancer towards standard chemotherapy show that this entity belongs to the tumor types, where women can benefit from cytotoxic treatment. Therefore, further improving treatment strategies in breast cancer might be more promising than in other tumor types poorly responding the chemotherapy. For this reason, we used MDA-MB-231 breast cancer cells. This cell line reveals several features of an aggressive phenotype such as invasiveness and formation of metastasis *in vivo* and insensitivity to anticancer drugs.

In the present investigation, we demonstrated that a resistance phenotype could be induced in MDA-MB-231 cells. Up-regulation of the transcription factors NFκB and Ap-1 associated with increased expression of ant-apoptotic *bcl-2* and reduced expression of pro-apoptotic *bax* can be discussed as underlying mechanism of action. These results obtained *in vitro* correspond with the weak activity of artesunate in MDA-MB-231 xenograft tumors *in vivo*.

## Materials and Methods

### Cell Culture Conditions

We obtained the estrogen receptor negative cell lines MDA-MB-231 and MDA-MB-468 [15], [16] (referred to as 231 and 468 cells) from ATCC_LGC (Wesel, Germany). MDA-MB-231 cells injected into the mammary fat pad of nude mice result in the formation of tumors and distant metastases in lungs, brain, and lymph nodes of most mice [17], whereas MDA-MB-468 are less tumorigenic and do not form metastasis *in vivo*.

Cells were grown at 37°C in a humidified atmosphere of 5% CO_2_ in MEM (Eagle's) with Earle's salts supplemented with 5% heat inactivated fetal calf serum, 1% L-glutamine solution (200 mM), 1% sodium pyruvate solution (100 mM), non-essential amino acids and vitamins. All cell culture material was obtained from PAA (Cölbe, Germany). Medium was changed every two days.

### Treatment of Cells

Artesunate was obtained from Saokim Ltd (Hanoi, Vietnam). The drug was dissolved in sterile DMSO (SIGMA-Aldrich; Taufkirchen, Germany) as a 1 mM stock solution and stored at −20°C. For the use in cell culture sterile dilutions in culture media were prepared. The maximal dilution of DMSO in the cell cultures did not exceed 1∶100. Therefore, toxic effects by DMSO can be excluded.

### Preparation of Conditioned Media

Cell culture supernatants of artesunate and carrier-treated-treated MDA-MB-231 cells were collected and centrifuged 15 min at 4000×g. The supernatants were used for Western Blots and zymography analyses.

### Determination of Protein Concentration

Protein concentrations were determined by the BCA protein assay (Pierce, Oud-Bejierland, Netherlands) with bovine serum albumin as standard.

### Preparation of RNA and cDNA synthesis

Total RNAs were isolated from cells treated with artesunate for several time periods using the RNeasy Mini Kit (Qiagen, Hilden, Germany) according to the manufacturer's instructions. Thereafter, oligo dT primed cDNAs were synthesized using the SuperScript® III First-Strand Synthesis SuperMix (Invitrogen, Irvine, CA) following the manufacturer's instructions.

### Quantitative RT-PCR

Expression analysis of a variety of genes was performed by quantitative real-time RT-PCR. All primers for the genes tested were designed using primer3 software [18] with a Tm optimum of approximately 60°C and a product length of 100–150 nt (see primer list, Table S1). Real time PCR was performed on an I-Cycler (Biorad Hercules, CA) using iQ Supermix (Biorad) supplemented with 10 nM fluorescein (Biorad), 0.1× SYBR-Green I (Sigma-Aldrich), 2.5 µL of cDNA (5× diluted), 3 pmol sense and antisense primers in a final reaction volume of 25 µL. After an initial denaturation step of 3 min during which the well factor was measured, 40 cycles of 15 sec at 95°C followed by 30 sec at 60°C were performed. Fluorescence was measured during the annealing step in each cycle. After amplification melting curves with 80 steps of 15 sec and 0.5°C increase were performed to monitor amplicon identity. Amplification efficiency was assessed for all primer sets utilized in separate reactions, and primers with efficiencies >94% were used. Expression data were normalized on HPRT, GAPDH and on RNA polymerase II (RPII) gene expression data obtained in parallel using the software BestKeeper [19]. Relative expression values with standard errors and statistical comparisons (unpaired two-tailed t-test) were obtained using Qgene software [20].

### Western Blots

Conditioned media from artesunate treated (3, 5, 7, 15, or 24 h) and non-treated control cells were analyzed using antibodies against MMP-1 (kind gift from Ralf Lichtinghagen, Medical School, Hannover) as previously described [21]. Enhanced chemiluminescence was used for visualization of the protein bands as recommended by the manufacturer (GE Healthcare, Little Chalfont, U.K.). Semiquantitative evaluation of the bands was performed by densitometric analysis with the ImageJ software provided by the NIH (http://rsb.info.nih.gov/ij/).

### MTT assay

The anti-proliferative effects of artesunate on MDA-MB-231 and MDA-MB-468 breast carcinoma cell lines were determined by the MTT (3-[4,5-dimethylthiazol-2-yl]-2,5-diphenyltetrazolium bromide) dye uptake method as previously described [22]. Pretreatment with artesunate was performed for 24 h by adding 20 µM of the drug to the culture media. Afterwards cells were washed 3 times with PBS and incubation with different concentrations of artesunate (2, 5, 10, 20, or 50 µM) was continued for another 24 h.

### Apoptosis Assay

Apoptotic cell death was determined by an enzyme-linked immunoassay (Cell Death Detection ELISA^PLUS^, Roche) to detect fragmented DNA and histones (mononucleosomes and oligonucleosomes). Human breast cancer cells MDA-MB-231 were seeded on 24-well plates and pretreated with 20 µM artesunate or carrier for 24 h. Afterwards cells were washed with PBS and treated with the carrier alone or different concentrations of artesunate in for another 24 h. Lysates prepared from the cells were processed following the instructions of the manufacturer.

The breast cancer cell lines MDA-MB-231 and MDA-MB-468 were grown in 24-well plates and incubated with Artesunate as described in the result section. The cells were harvested and treated with (FITC)-conjugated annexin V and propidium iodide (Annexin-V-FLUOS Staining kit from Roche Diagnostics (Mannheim, Germany) according to the recommendations of the manufacturer. Ten thousand events were counted for each sample. Data were analyzed using a Flow-Cytometer (Beckmann Coulter XL-MCL, Software: System II).

### Electrophoretic Mobility Shift and Supershift Assays

Cells were seeded onto 150 cm^2^ culture dishes with 25 mL culture medium and treated with artesunate or the carrier alone for several time periods (2, 4 or 6 h). Nuclear extracts were prepared as previously described [23]. Oligonucleotides corresponding to the consensus sequences (AP-1 site: 5′-GAT CTG TGA CTC AGC GCG AG-3′; NFκB site: 5′-GTT AGT TGA GGG GAC TTT CCC A-GGC-3′) were labeled with [^32^P]dATP (3000 Ci/mM) and Klenow enzyme and were incubated with 10 µg of nuclear protein in 20 µL of 7 mM Hepes-KOH (pH 7.9), 100 mM KCl, 3.6 mM MgCl_2_, and 10% glycerol on ice for 20 min. Poly[d(I-C)] (0.05 mg/mL) was added as an unspecific competitor. The samples were run on a 5% non-denaturing polyacrylamide gel in a buffer containing 25 mM Tris-HCl (pH 8.0), 190 mM glycine, and 1 mM EDTA. Gels were dried and analyzed by autoradiography. In order to prove the specificity of the probes, a 50-fold excess of unlabeled probe was incubated with the binding reaction mixture for 45 min on ice before adding the radiolabeled DNA fragment. Specificity of binding ascertained by Superhift was performed using a specific antibodies against the p65 subunit of NFκB and c-jun (Santa Cruz, USA).

### Casein Zymography

Gelatin zymography was essentially performed as previously described in detail [24]. A molecular mass standard (Biorad, Germany) was used in all experiments. These experiments were repeated three times.

### Promoter analysis

Promoter sequences were analyzed for the presence of transcription factor binding sites using oPossum (www.cisreg.ca/oPOSSUM; [25]. Sequence 5000 nts upstream and 2000 nts downstream of the transcription start site were considered, the matrix match threshold was set to 85%.

### Data analysis

Statistical significance was assessed by comparing mean (±SD) values, which were normalized to the control group with Student's t-test for independent groups. One-way analysis of variance was used to test for statistical significance (*P*<0.05), and when significance was determined, Bonferroni's multiple comparison test was performed post hoc, as indicated in the figure legends. Statistical analysis was performed using the Prism software (GraphPad, San Diego, CA).

### Animal Experiments

Animals: For the animal experiments, female nude mice (Ncr∶nu/nu), ages 4 to 6 weeks and weighing 20–24 g, were used as described earlier [26]. All animal experiments were performed according to the German Animal Protection Law and by approval of the local responsible authorities (Approval Number: GV247/98; Ethics Committee of the *Landesamt für Gesundheit und Soziales*, Berlin, Germany).

Tumor Inoculation: Each mouse received 10^7^ tumor cells subcutaneously without anesthesia. The injected cells were diluted in isotonic salt solution and subcutaneously injected into the mice. The diameter of the tumors was measured twice weekly using a caliper-like mechanical instrument and the tumor volume (V) was calculated according to the empirical equation V = (length×width^2^)/2. The median volumes of each group were normalized to the initial tumor volume resulting in the relative tumor volume.

Treatment modalities: Each six MDA-MD-231-transplanted animals received artesunate (200 and 400 mg/kg, respectively) or vehicle (10% Tween80 in saline) intraperitoneally (i.p.) at five consecutive days. Treatment started when tumors were palpable (about 5–6 mm diameter). An additional group of xenografted animals were treated with doxorubicin (i.v., 8 mg/kg) once a week for two weeks.

## Results

### Acquired resistance in terms of cell viability

**Figure 1:**
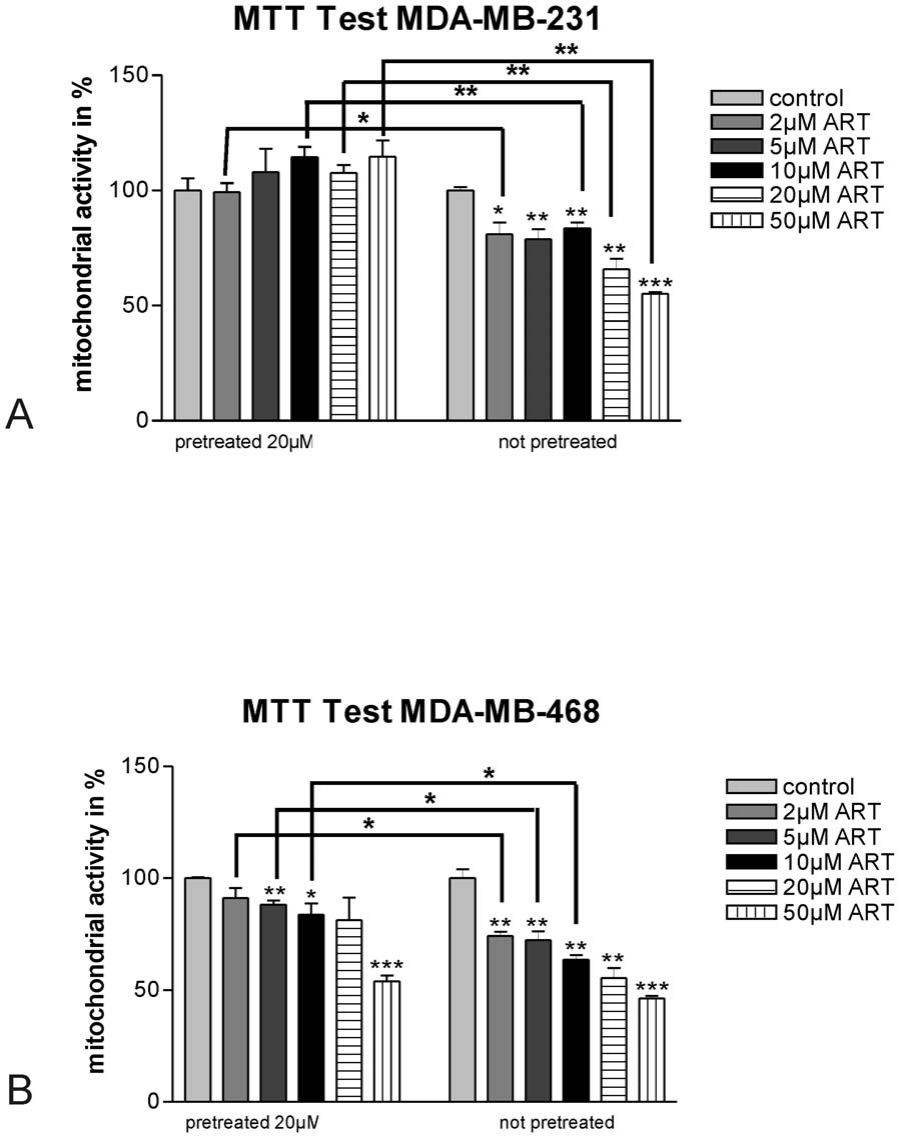
Cell viability. A) Different concentrations of artesunate reduce cell viability measured by MTT assay up to approximately 40% (50 µM) after 24 h in MDA-MB-231 cells (right panel), which was statistically significant (* *P*<0.05; ** *P*<0.01; *** *P*<0.001; one way Anova with Bonferroni's post test). In contrast, MDA-MB-231 cells pretreated with 20 µM artesunate for 24 h acquired resistance and did not longer respond to application of the drug at various concentrations for further 24 h (left panel). B) Human non-metastastc breast cancer cells MDA-MB-468 responded to artesunate treatment at various concentrations with a statistically significant decline in cell viability up to 50% (** *P*<0.01; *** *P*<0.001; one way Anova with Bonferroni's post test) (right panel). After 24 h pretreatment with 20 µM artesunate, MDA-MB-468 cells still responded to the drug with decreased cell viability down to about 55%, which was statistically significant (*** *P*<0.001; one way Anova with Bonferroni's post test).

As a first step, the effect of artesunate on the viability of the human breast cancer cell lines MDA-MB-231 and MDA-MB-468 was analyzed by MTT assays. Artesunate induced resistance in the highly metastatic MDA-MB-231 cells (Fig. 1A) and to a lesser extent also in non-metastatic MDA-MB-468 cells (Fig. 1B).

In comparison to carrier-treated cells, cell viability of MDA-MB-231 cells treated for 24 h with different concentrations of artesunate (2, 5, 10, 20 and 50 µM) declined in response to the dose applied to about 55% (50 µM) with high statistical significance: *P*<0.05 for 2 µM; *P*<0.01 for 5, 10 and 20 µM; *P*<0.001 for 50 µM (Fig. 1A, right panel).

When MDA-MB-231 cells were pretreated for 24 h with 20 µM artesunate, the cells did not respond to any further treatments showing that the cells acquired drug resistance (Fig. 1A, left panel).

MDA-MB-468 cells responded to application of artesunate and cell viability dropped in a dose-dependent manner to about 45% with statistical significance: *P*<0.01 for 2, 5, 10 and 20 µM; *P*<0.001 for 50 µM (Fig. 1B, right panel). In comparison to MDA-MB-231 cells, pretreatment of MDA-MB-468 cells with 20 µM artesunate for 24 h induced resistance to a lesser extent with a constant decline in cell viability in a dose-dependent manner to further application of the drug with statistical significance: *P*<0.05 for 10 µM, *P*<0.01 for 5 µM and *P*<0.001 for 50 µM (Fig. 1B, left panel). In contrast to the carrier-treated controls, cell viability dropped to further 55%.

### Acquired resistance in terms of apoptosis

**Figure 2:**
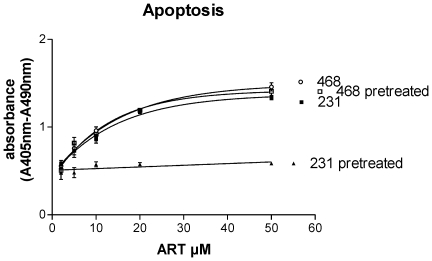
Detection of nucleosomes in cytoplasmic fractions (Cell Death ELISA, Roche Applied Biosystems) of MDA-MB-231 and MDA-MB-468 cells treated for 24 h with various concentrations of artesunate in comparison to the cells pre-treated for 24 h with 25 µM artesunate. While MDA-MB-468 cells undergo apoptosis upon artesunate treatment no matter if cells are pretreated for 24 h with artesunate (indicated with □) or not (indicated with ○), MDA-MB-231 cells acquired resistance to artesunate treatment and do not undergo apoptosis (indicated with ▪ or ▴ respectively) when pretreated for 24 h with artesunate.

Apoptosis measured by means of increased nucleosomes in cell lysates was induced in MDA-MB-231 and MDA-MB-468 cells in a dose-dependent manner after 24 h treatment (Fig. 2 (▪)). Thereby, the apoptosis rate was increased about 2-fold by 20 µM and about 3-fold by 50 µM artesunate. Pretreatment of MDA-MB-231 and MDA-MB-468 cells with 20 µM ART for 24 h induced resistance to apoptosis in the cells and likewise the number of nucleosomes in lysates of the pretreated cells did not increase upon further treatment with various doses of artesunate (Fig. 2 (▴)).

**Figure 3:**
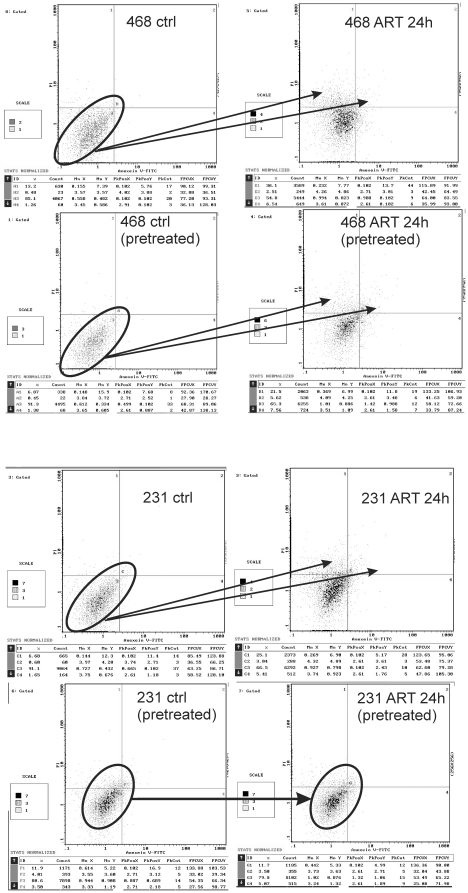
Flow cytometry analysis of early and late apoptosis in non-resistant MDA-MB-468 and resistant MDA-MB-231 cells. Evidence of early apoptosis in terms of translocation of phospholipid phosphatidylserine from the inner to the outer leaflet of the plasma membrane, where it becomes accessible to annexin V staining, can be seen in quadrants 4 and 2. Cells reaching a late apoptotic propidium iodide positive state can be seen in quadrant 1. MDA-MB-468 cells treated with ART for 24 h, showed clear evidence of early and late apoptosis, no matter if they were pretreated for 24 h with ART. The two arrows indicate the shift from the lower left quarter to the upper right and upper left quarters. In contrast ART treatment induced early and late apoptosis only in non pretreated MDA-MB-231 cells, whereas pretreated MDA-MB-231 cells did not further undergo early or late apoptosis as illustrated by the two circles and the arrow in the lower panel of the figure.

In order to confirm our results obtained by measuring the increase of nucleosomes and to clearly distinguish between early and late apotosis, we performed flow cytometry analysis of apoptosis and necrosis in non-resistant MDA-MB-468 and resistant MDA-MB-231 cells (Fig. 3). MDA-MB-468 cells treated with ART for 24 h, showed clear evidence of early and late apotosis, no matter whether they were pretreated for 24 h with ART. In contrast, ART treatment induced early and late apotosis only in non-pretreated MDA-MB-231 cells, whereas pretreated MDA-MB-231 cells did not further undergo early and late apotosis.

### Activation of NFκB and AP-1 (EMSA) by artesunate

In order to unravel the molecular mechanism of acquired resistance in MDA-MB-231 cells, we analyzed the effect of artesunate on the transcription factors NFκB and AP-1. In case of nuclear activation of these factors, apoptosis is down-regulated due to over-expression of cell survival related genes and repression of pro-apoptotic genes.

**Figure 4:**
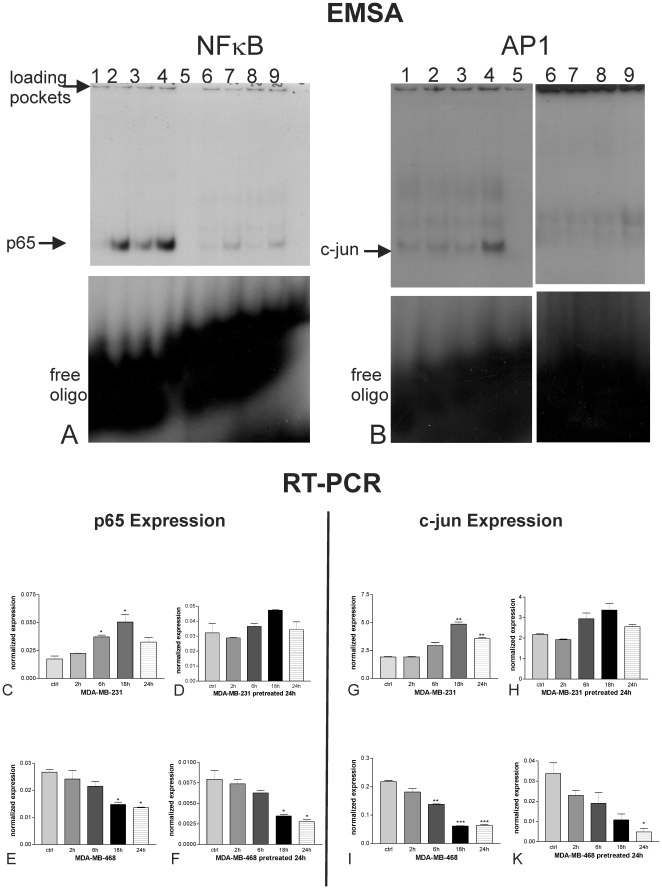
Effects of artesunate on NFκB (A) and AP-1 (B). Binding of ^32^P-labeled NFκB and AP-1 specific oligonucleotides to the cognate transcription factor present in nuclear extracts of MDA-MB-231 cells was monitored by EMSA. Artesunate treatment of MDA-MB-231 cells for different time intervals (lane 2, 2 h; lane 3, 4 h; lane 4, 6 h) led to a induced binding of NFκB (A) and AP-1 (B) to its response element as compared to carrier-treated cells (lane 1). The specificity of the binding was assessed for all three oligos by addition of a 50× molar excess of cold oligonucleotides (lanes 5). Furthermore, addition of appropriate specific antibodies(supershift) against p65 or c-jun respectively resulted in the formation of very faint or rather diffuse double bands (lanes 6–9). Experiments were repeated at least three times. Fig. 4 C-K illustrates the expression of p65 and c-jun in MDA-MB-231 and -468 cells. mRNA expression of p65 was induced in MDA-MB-231 cells upon artesunate treatment only in non-pretreated cells (C), while p65 expression did not alter upon artesunate treatment in pre-treated cells (D). MDA-MB-468 cells p65 expression was down-regulated statistically significantly after 18 h and 24 h upon artesunate treatment, no matter whether the cells were pretreated with artesunate (F) or not (E). The effect of acquired resistance as seen for p65 expression could also be found concerning the expression of c-jun. While c-jun was statistically significantly up-regulated in MDA-MB-231 cells after 18 h and 24 h, no significant change could be observed for cells pretreated 24 h with artesunate. (* P<0.05; ** P<0.01; *** P<0.001; one way Anova with Bonferroni's post test). Experiments were performed in triplicates.

Nuclear extracts from artesunate-treated and carrier-treated cells were subjected to electrophoretic mobility shift assays (EMSA), where the binding of transcription factors is revealed by the retarded electrophoretic migration of radioactively labeled oligonucleotides of the specific binding sequence. Binding of the transcription factors NFκB and AP-1, which are both associated with resistance to standard anti-cancer drugs, was monitored (Fig. 4A and B). Artesunate treatment for 2, 4, and 6 h of MDA-MB-231 cells (lanes 2, 3, and 4, respectively) showed a clear induction in the specific bands for the NFκB transcription factor subunit p65 (Fig. 4A) and the AP-1 subunit *c-jun* (Fig. 4B) in comparison to carrier-treated cells (lanes 1). The specificity of the binding was assessed by addition of a 50-fold excess of cold oligonucleotides that abolished the band shifts observed (lanes 5). Furthermore, the bands disappeared upon addition of appropriate specific antibodies (supershift) against p65 or c-jun respectively. The interference of the antibodies with the binding of the proteins (transcription factors) to the labelled probes resulted in the formation of very faint or rather diffuse double bands (lanes 6–9). Addition of an unrelated mutant oligonucleotide had no effect on NFκB binding (data not shown). Experiments were repeated at least three times.

Expression of the NFκB subunit p65 (Fig. 4C–F) and the AP-1 subunit *c-jun* (Fig. 4G–K) were analyzed by quantitative RT-PCR. mRNA expression of p65 was induced in MDA-MB-231 cells upon treatment throughout 24 h, whereby the peak of induction was reached after 18 h (* P<0.05; one way Anova with Bonferroni's post test). After pre-treatment of MDA-MB-231 cells with ART for 24 h, no statistically significant changes in p65 expression could be observed. In non-resistant MDA-MB-468 cells p65 expression is down-regulated statistically significantly (* P<0.05; one way Anova with Bonferroni's post test) after 18 h and 24 h upon ART treatment no matter whether the cells were pretreated with ART or not. The effect of acquired resistance as seen for p65 expression could also be found concerning the expression of c-jun. While c-jun was statistically significantly (** P<0.01; one way Anova with Bonferroni's post test) up-regulated in MDA-MB-231 cells after 18 h and 24 h, no significant change could be observed for cells pretreated 24 h with ART. Again MDA-MB-468 cells responded always to ART treatment with down-regulation of c-jun expression, which was statistically significant at least after 24 h no matter if the cells were pretreated with ART or not.

Our results show that human metastatic breast cancer cells MDA-MB-231 acquired resistance to ART treatment and thereby expression of p65 and c-jun do not alter upon further treatment with the substance.

Acquired resistance becomes also clear by comparing the percentage of cells undergoing early and late apotosis upon further ART treatment in pretreated or non pretreated breast cancer cells (Table 1). While MDA-MB-468 cells have a significant increase in early (6.18%) and late apoptosis (14.63%), MDA-MB-231 cells showed only a minimal increase in early apoptosis (2.29%) and no increase in late apoptosis 24 h after additional ART treatment.

**Table 1:** Percentage of cells undergoing apoptosis or necrosis.

	MDA-MB-468				MDA-MB-231			
			ART pretreatment				ART pretreatment	
	ctrl	24 h ART	ctrl	24 h ART	ctrl	24 h ART	ctrl	24 h ART
Early apoptosis	1.26	6.54	1.38	7.56	1.65	5.41	3.58	5.87
Late apoptosis	13.2	36.1	6.87	21.5	6.58	25.1	11.9	11.7

Early and late apoptosis measured by annexin V and propidium iodide staining in human breast cancer cells revealed that the metastatic MDA-MB-231 cells pretreated for 24 h with artesunate, became resistant to further treatment. While MDA-MB-468 cells showed significantly increased early (6.18%) and late apoptosis (14.63%), MDA-MB-231 cells showed only a minimal increase in early apoptosis (2.29%) and no increase in late apoptosis 24 h after additional artesunate treatment.

### Bcl-2 and bax are involved in the development of resistance

As recent evidence accumulates that the ratio between *bcl-2* and *bax* is of special interest for the induction of apoptosis in cancer cells, we examined the expression of these two members of the *bcl-2*-family, in order to evaluate their role in the development of resistance against artesunate.

**Figure 5:**
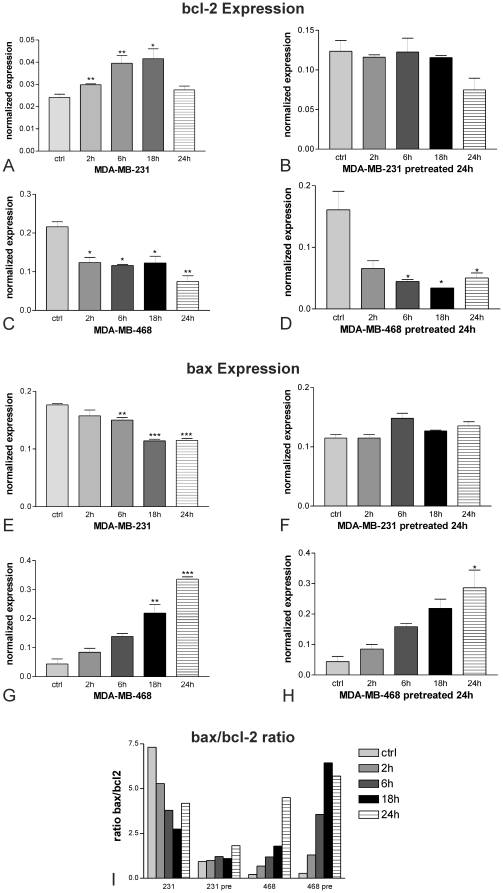
Expression of apoptosis-related genes as determined by RT-PCR. (A–D) Treatment with 20 µM leads to induction of *bcl-2* expression in MDA-MB-231 cells after 2 h (** *P*<0.01) and reaches a level of about two fold after 18 h with statistical significance of * *P*<0.05 in comparison to carrier-treated control cells (A). Pretreatment with artesunate rendered MDA-MB-231 cells resistant to this compound and, hence, bcl-2 expression could not be induced in these cells (B). In contrast, pretreatment with artesunate did not lead to resistance in MDA-MB-468 cells. Consequently, bcl-2 expression was statistically significantly diminished in these cells already after 6 h with further decline up to 24 h, no matter if cells were pretreated (C) or not (D). (E–H) Expression of the pro-apoptotic factor *bax* was repressed in MDA-MB-231 cells upon treatment between 6 and 24 h(E). As already seen for bcl-2 expression, pretreatment with artesunate rendered MDA-MB-231 cells resistant and, therefore, bax expression could not be inhibited by artesunate in these cells (F). On the other hand, expression of bax in MDA-MB-468, which did not acquire resistance against artesunate, could be induced by the compound, already after 2 h with further significant increase up to 24 h, no matter whether cells were pretreated or not. Experiments were performed in triplicates (* P<0.05; ** P<0.01; *** P<0.001; one way Anova with Bonferroni's post test). (I) Since apoptosis results from the balance of pro- and anti-apoptotic members of the bcl-2 family, we calculated the expression ratios of the pro-apoptotic bax and the anti-apoptotic bcl-2 as indicator for induction or repression of apoptosis by artesunate. High ratios indicate cellular proficiency to induce apoptosis, while low ratios may occur in more apoptosis-resistant cells.

Treatment of the human metastatic breast cancer cell line MDA-MB-231 with 20 µM artesunate, led to induction of *bcl-2* expression already after 2 h (** *P*<0.01). Up-regulation of this anti-apoptotic factor reached a level of about two fold after 18 h with statistical significance of * *P*<0.05 in comparison to carrier-treated control cells (Fig. 5A). Pretreatment with ART rendered MDA-MB-231 cells resistant to this compound and hence bcl-2 expression could not be induced in these cells (Fig. 5B). In contrast, pretreatment with ART did not lead to resistance in MDA-MB-468 cells. Consequently, bcl-2 expression was diminished in these cells already after 2 h with further decline up to 24 h, no matter whether cells were pretreated (Fig. 5C) or not (Fig. 5D).

Expression of the pro-apoptotic factor *bax* was repressed in MDA-MB-231 cells upon artesunate treatment. This anti-apoptotic effect became statistically significant after 6 h with ongoing decline up to 24 h with a significance of *** *P*<0.001 in comparison to carrier-treated cells (Fig. 5E). As already seen for bcl-2 expression, pretreatment with ART rendered MDA-MB-231 cells resistant and, therefore, bax expression could not be inhibited by ART in these cells (Fig. 5F). On the other hand, expression of bax in MDA-MB-468, which did not acquire resistance against ART, could be induced by the compound, already after 2 h with further significant increase up to 24 h, no matter whether cells were pretreated (Fig. 5H) or not (Fig. 5G).

Apoptosis results from the balance of pro- and anti-apoptotic members of the bcl-2 family. Therefore, we calculated the ratio of the pro-apoptotic bax and the anti-apoptotic bcl-2 as indicator for induction or repression of apoptosis by artesunate. High ratios indicate cellular proficiency to induce apoptosis, while low ratios may occur in more apoptosis-resistant cells. As shown in Figure 5 (I), the bax/bcl-2 ratio was high in non-pretreated MDA-MB-231 cells and decreased over time, indicating that the cells acquired resistance to artesunate-induced apoptosis. In contrast, the bax/bcl-2 ratio was low in pretreated cells and did not increase up to 18 h and only a little bit after 24 h. this may indicate that the cells remained apoptosis-resistant towards artesunate. The strongly increasing bax/bcl-2 ratios in both pretreated and non-pretreated MDA-MB-468 cells indicate that artesunate pretreatment did not result in apoptosis-resistance towards artesunate.

The correlation between the induction of the transcription factors NFκB and the regulation of the apoptotis-related genes *bcl-2* and *bax* was confirmed by promoter analysis. Both genes share all of the transcription binding factor sites identified by high stringency analysis of the promoter region from −5000 to +2000 (ELK4,ELF5, SPIB, NFκB, ZNF354C, SP1, ELK1, MZF1_1–4, MZF 5–13, Bapx1) indicating a common regulation. *bax* had a *bona fide* NFκB in position −363 (sequence: GGGCCTGCCC) and *bcl-2* had two of binding sites in position −641 and +139 (sequences: GGCAATTTAC), relative to the transcription start site (Table 2).

**Table 2:** Analysis of NFκB binding sites in the apoptosis genes.

Promoter Analysis						
Gene	ENSEMBL ID	Chr.	Transcription factor binding site	Position relative to transcription start	Orientation	Z-Score
BAX	ENSG00000087088	19	GGGCCTGCCC	354	−1	7.01e+00
BCL2	ENSG00000171791	18	GGGACTTCCA	3745	−1	9.80e+00
			GGGACTTCCA	2966	−1	9.80e+00
			GGCAATTTAC	650	1	7.04e+00
			GGCAATTTAC	−130	1	7.04e+00

The promoter regions of BAX and BCL2 from −5000 to +2000 relative to the transcription start site were analyzed for the occurrence of NFκB binding sites using the Opossum web-service. The identity of the sequence used for analysis (ENSEMBL ID), the chromosome number (Chr.), the actual transcription factor binding site, the position relative to the transcription start site and the orientation are indicated. The Z-score uses the normal approximation to the binomial distribution to compare the rate of occurrence of a TFBS in the target set of genes to the expected rate estimated from the pre-computed background set. The likelihood that the number of TFBS nucleotides detected for the included target genes was significant as compared with the number of TFBS nucleotides detected for the background set. Z-score was expressed in units of magnitude of the standard deviation.

### Effect of artesunate on expression and activity of MMP-1 in resistant breast cancer cells

**Figure 6:**
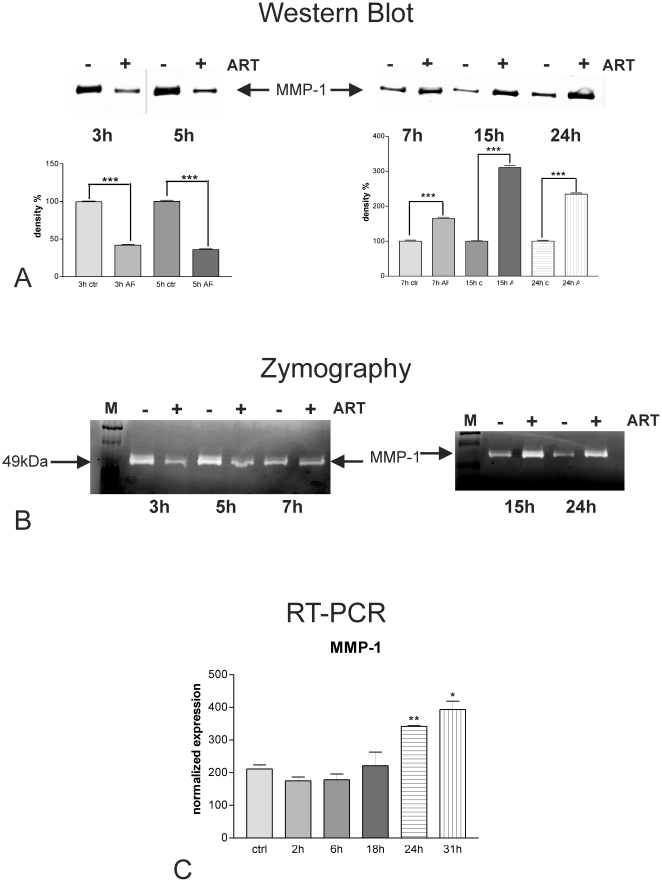
Effect of artesunate on MMP-1 expression and activity. A) Western Blots of conditioned media from MDA-MB-231 cells treated with 20 µM artesunate for several time periods (lanes indicated with “+”) in comparison to carrier-treated control cells (lanes indicated with “−”) using specific antibodies against MMP-1 reveal that artesunate inhibited MMP-1 secretion when applied for shorter time periods (3 h, 5 h, left panel), while it induced MMP-1 when applied for longer time periods (7, 15 and 24 h, right panel). According to densitometrical analysis of the bands (lower panels), down-regulation of MMP-1 protein secretion in response to artesunate treatment, seen for early time points (3 and 5 h, lower left panel), was about 60–65% and up-regulation was about 1.6-fold after 7 h, 3-fold after 15 h and 2.3-fold after 24 h of artesunate treatment (lower right panel). *** *P*<0.001 (one way Anova with Bonferroni's post test). B) Casein Zymography (analysis of MMP-1 activity) was in accord with the results obtained from Western Blot analysis and showed a reduction of proteolytic activitiy present in conditioned media from MDA-MB-231 cells treated for 3 and 5 h with 20 µM artesunate (ART) (lanes indicated with “+”) as compared to those of carrier-treated cells (lanes indicated with “-“). In contrast, cells treated for longer periods (15 and 24 h) with artesunate (lanes indicated with “+”) showed a clear induction of MMP-1 as depicted here in Fig. 5A, right panel. Interestingly, treatment with artesunate for 7 h did not result in any differences in MMP-1 secretion into the culture medium of MDA-MB-231 cells when compared to carrier-treated cells (lanes indicated with “−”). MMP-1 was identified according to its migration behavior as compared to a molecular mass standard (lanes indicated with M). C) Quantification of MMP-1 mRNA expression by real time RT-PCR of MDA-MB-231 breast cancer cells treated with 20 µM artesunate for several time periods revealed a statistically significant induction of MMP-1 only after 24 up to 31 h treatment. Shorter treatment periods did result in a slight reduction of MMP-1 transcripts. Experiments were performed in triplicate; error bars indicate SD; * *P*<0.05; ** *P*<0.01 (student's t-test).

We analyzed the amount of the metastasis-related protease MMP-1 secreted into the cell supernatants of artesunate treated human breast cancer cells MDA-MB-231 by Western blots with specific antibodies against MMP-1 (Fig. 6A). Thereby, accumulation of newly released MMP-1 secreted into fresh serum-free medium added at the beginning of the time course was monitored in the presence (+) or in the absence (−) of 20 µM artesunate. We found that MMP-1 protein was released into the supernatants of MDA-MB-231 cell cultures. For shorter time periods (3 and 5 h, left upper panel), the amount of MMP-1 protein secreted into the media of treated cells was lower compared to those of non-treated cells. After 7 h of artesunate treatment, the effect switched and supernatants of treated cells had elevated MMP-1 concentrations in comparison to non-treated breast cancer cells. Up-regulation of MMP-1 secretion was observed for up to 24 h artesunate treatment (7, 15 or 24 h, upper right panel). All experiments have been performed in triplicates. According to determination of protein concentrations by Pierce assay equal amounts of secreted protein were subjected to SDS-PAGE, and as loading control, the amount of protein blotted onto the membranes was visualized with Ponceau red before blocking (data not shown). Densitometric analysis of the bands (Fig. 6A, lower panels) revealed that the down-regulation of MMP-1 protein secretion in response to artesunate treatment, seen for early time points (3 and 5 h; Fig. 6A, lower left panel), was about 60–65%. Up-regulation of MMP-1 secretion was about 1.6-fold after 7 h, 3 fold after 15 h and 2.3-fold after 24 h of artesunate treatment (Fig. 6A, lower right panel). The statistical power for all observed regulation events was *P*<0.001 throughout the whole experiments (one way Anova with Bonferroni's post test).

In order to visualize the enzymatic activities present in cell culture supernatants, we performed zymography analyses on casein-containing gels (Fig. 6B). As MMP-1 is able to degrade casein, electrophoretically separated proteases, present in cell culture supernatants, were detected as translucent bands on the Coomassie brillant blue stained substrate background.

MDA-MB-231 breast cancer cells responded to the artesunate treatment (lanes labeled with “+”) with a transient (3 and 5 h, left panel) reduction of MMP-1 activity released into fresh serum-free medium in comparison to carrier-treated cells (lanes labeled with “−”). In contrast, cells treated for longer periods (15 h and 24 h) with artesunate (+) showed a clear induction of MMP-1 as depicted here in Fig. 6A, right panel. Interestingly, treatment with artesunate for 7 h did not result in any differences in MMP-1 secretion into the culture medium of MDA-MB-231 cells when compared to carrier-treated cells (−). MMP-1 was identified according to its migration behavior as compared to a molecular mass standard (lanes labeled with M).

In order to monitor the effect of artesunate on the level of MMP-1 transcription, we performed quantitative Real Time RT-PCR (Fig. 6C) and normalized expression values on those obtained for the housekeeping genes RPII and HPRT. In accordance with the results from Western blots and zymography, shown in the sections before, artesunate treatment of MDA-MB-231 cells resulted in slightly diminished levels of MMP-1 mRNA after 2 and 6 h. However, after 18 h treatment the reduction of MMP-1 mRNA expression by artesunate faded out and treatment with 20 µM of the drug for longer time periods led to statistically significant inductions of MMP-1 of 1.6- and 1.9-fold after 24 and 31 h respectively. The levels of statistical significance were *P*<0.01 at 24 h and *P*<0.05 at 31 h (student's t-test).

### Activity of artesunate towards MDA-MB-231 xenograft tumors in nude mice

**Figure 7:**
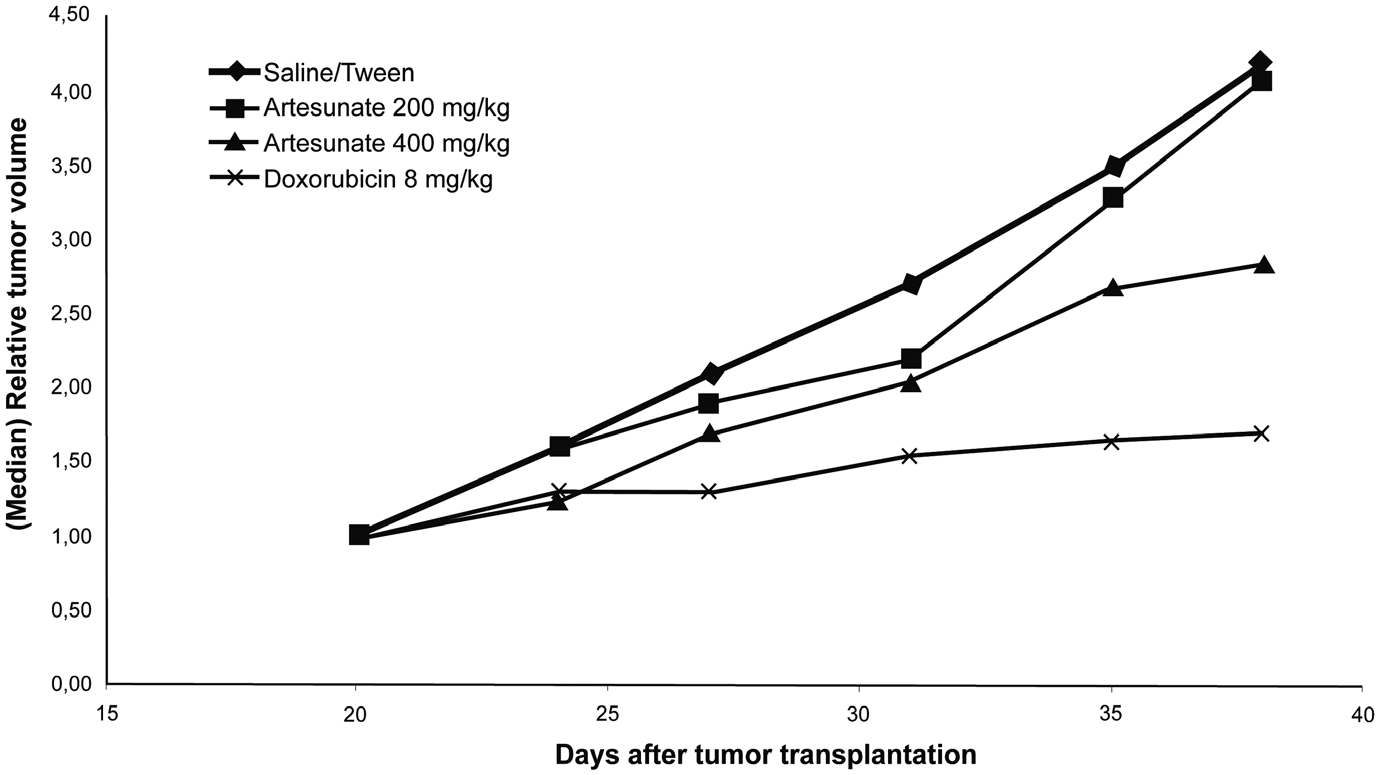
Activity of artesunate towards MDA-MB-231 xenograft tumors in nude mice. Xenografted mice were either treated i.p. with vehicle (10% Tween 80 in saline) or with 200 and 400 mg/kg artesunate, respectively, at five consecutive days. In addition, a control group was treated with doxorubicin 8 mg/kg i.v.) twice in a weekly distance. The relative tumor volume over time is shown of each six animals per treatment group.

The anti-tumor activity of artesunate was examined *in vivo* in MDA-MB-231 xenografted tumors in nude mice. The results are shown in Fig. 7. While treatment with 200 mg/kg artesunate did not result in inhibition of tumor growth as compared to vehicle-treated tumors, a marginal inhibition was observed with 400 mg/kg artesunate. By contrast, a considerable inhibition of tumor growth was reached using a control drug, doxorubicin. We did not observe any signs of toxicity of artesunate (loss of weight etc.).

## Discussion

In the present investigation, we showed that sub-lethal doses of artesunate resulted in the development of resistance towards this drug. While inherent differences of cancer cell lines in response towards artesunate and modulation of inherent resistance by ferrous iron were previously described [4], [27], the present study is to the best of our knowledge the first to show an acquired resistance phenomenon towards artesunate in cancer cells. The induced resistance observed *in vitro* corresponds to the unresponsiveness at lower artesunate doses (200 mg/kg) and only minor responsiveness after treatment with high artesunate doses (400 mg/kg) after repeated injection of the compound to nude mice as shown here.

Recently, it was reported that artemisinin treatment can confer resistance to other anti-cancer drugs such as doxorubicin [28]. Resistance towards artesunate-type drugs occurs only infrequently in malaria parasites, but has been reported both in patient samples and under *in vitro* conditions [12], [13], [14]. The role of several plasmodial proteins, including multidrug resistance transporters (pfmdr1), sarcoendoplasmatic Ca^2+^-dependent ATPase (SERCA), translationally controlled tumor protein (TCTP) and others have been discussed.

Here, we present evidence that MDA-MB-231 human breast cancer cells reveal reduced sensitivity after repeated artesunate treatment. Likewise diminished cytotoxicity was seen in pre-treated highly metastatic MDA-MB-231 cells in contrast to less tumorigenic non-metastatic MDA-MB-468 breast cancer cells. This observation implicates that the drug could be beneficially applied as therapy in less advanced breast cancer. Additionally apoptosis was down-regulated in metastatic breast cancer cells pre-treated with the drug for 24 h.

Analysis of molecular mechanisms underlying the acquired resistance against ART in the breast cancer cells showed that this was due to activation of the transcription factors, NFκB and AP-1. NFκB represents a central player for many cellular processes such as proliferation, adhesion, angiogenesis, inflammation and others. It mediated apoptosis resistance towards various stimuli, including anti-cancer agents and is activated by DNA damage [29].

NF-κB is a resistance factor for established anticancer drugs by inhibiting apoptosis [30], [31]. Recently, we have shown that artesunate induces double stand breaks [32]. In non-activated cells, NFκB is complexed with IκB in the cytosol. Upon stimulation by appropriate stimuli, the complex is dissociated and NFκB translocates into the nucleus, where it binds to specific promoters of target genes, *e.g.* survival-related genes, and stimulates their transcriptional activation.

The AP-1 complex consists of c-Fos, FosB, Fra-1, or Fra-2, each of which can dimerize with c-Jun, JunB, or JunD. This complex binds to specific binding motifs in the promoter of target genes regulating, apoptosis, proliferation, or differentiation. A role for AP-1 for resistance to anti-cancer drugs has also been proposed [33]. In a previous investigation, we found that AP-1 (together with Sp1) was activated in host cells upon infection with human cytomegalovirus and that a single artesunate treatment suppressed AP-1 activation and production of virus-specific proteins in host cells [34]. AP-1 acts as transcription factor for anticancer drug resistance genes such as P-glycoprotein/*MDR1* or glutathione S-transferase-pi. Glutathione S-transferases detoxify harmful xenobiotic molecules by binding them to glutathione. Then, glutathione-drug conjugates are transported out of cells. The expression of glutathione S-transferases was also associated with cellular response of tumor cells to artesunate [35], [36].

Here, we observed that repeated treatment of highly metastatic breast cancer cells induced AP-1 activation leading to artesunate resistance. Whether a similar phenomenon can also be found in anti-viral therapy with artesunate is unknown yet.

Similar to AP-1, we found an activation of NFκB associated with development of artesunate resistance in MDA-MB-231 cancer cells. In immune cells, artesunate-type drugs have been described to inhibit NFκB activation causing immunomodulatory effects [37], [38], [39]. On the other hand, NFκB activation has been reported as potential mechanism of gemcitabine resistance in MCF-7 and MDA-MB-231 breast cancer cells [40]. These results are comparable to our here presented data on artesunate resistance in MDA-MB-231 cells.

Both, NFκB and AP-1 are known to regulate apoptosis-regulating *bcl-2* family members [41]. Dimerization of *bax* with other pro-apoptotic *bcl-2* family members leads to the formation of mitochondrial membrane pores enabling cytochrome C release into the cytosol and onset of cell death. Anti-apoptotic *bcl-2* family members inhibit pore formation and cytochrome C release by binding to *bax*.

The identification of multiple *bcl-2* homologues many of which form homo- or heterodimers suggests that these molecules function at least in part through protein-protein interactions. *bax* heterodimerizes with *bcl-2* and homodimerizes with itself. When *bax* is over-expressed in cells, apoptotic death in response to a death signal is accelerated. When *bcl-2* is over-expressed, it heterodimerized with *bax* and death is repressed [42]. Thus, the ratio of *bcl-2* to *bax* is important in determining susceptibility to apoptosis.

Therefore, we analyzed the expression of the pro-apoptotic and anti-apoptotic genes after treatment with artesunate. The expectation was that expression levels of pro-apoptotic genes will decrease and of anti-apoptotic genes will increase during development of resistance. *Bcl-2* expression increased upon challenge with artesunate. This indicates that *bcl-2* may have a specific function for induction of artesunate resistance. Among the pro-apoptotic genes, *bax* expression decreased after artesunate treatment suggesting that down-regulation of *bax* participated in development of artesunate resistance. The stable expression of bax at 18 and 24 h may be explained by a rheostat of anti-and pro-apoptotic bcl-2 family members, of which bax is only one pro-apoptotic factor. It cannot be excluded that other pro-apoptotic members are also down-regulated supporting the function of bax. Functional analysis of apoptosis by detection of nucleosomes in cytoplasmic fractions of MDA-MB-231 cells treated with various concentrations of artesunate in comparison to MDA-MB-231 cells pre-treated with artesunate revealed that artesunate induced apoptosis only in cells that have not been pre-treated. These results strongly suggest that MDA-MB-231 cells acquire resistance against cell death. *Bcl-2* is up-regulated and *bax* is down-regulated upon treatment with artesunate, though transiently. However, both share similar TF-binding sites, especially sites for NFκB. The differential regulation can therefore not simply be explained by enhanced NFκB activity following ART treatment. *bax* is also under control of p53 which might be induced by artesunate induced double strand breaks. MDA-MB 231 cells have a mutation in exon 5 of the p53 gene (www-p53.iarc.fr) leading to an almost non functional transcriptional activity conserving, however, some activity on the *bax* promoter [43]. It is conceivable that NFκB has an immediate survival effect mediated by *bcl-2* with a potential feedback via *bax*, with the latter being dependent on other factors such as p53. Moreover, *bcl-2* has two bona fide binding sites and *bax* only one; kinetics or the strength of induction might therefore vary between the two genes. It should however be taken into account that NFκB affects other signaling routes (*e.g.* JNK phosphorylation) as well to regulate drug-induced apoptosis [44].

The human metastatic breast cancer cells MDA-MB-231 are a well-known model for studying tumor aggressiveness, invasion and metastasis. Matrix metalloproteinases (MMP), especially MMP-1 that is able to degrade fibrillar native collagen type I, are involved in the metastatic process, and also in the inhibition of apoptosis and drug resistance [45], [46]. Likewise it has been shown that multidrug resistant cell lines produced more MMP-1, -2 and -9 compared to their analogous non resistant cells [47].

Therefore, we also analyzed the effect of artesunate on MMP expression and observed that for short treatment periods artesunate transiently down-regulates MMP-1 on the levels of expression, secretion and enzymatic activity in metastatic MDA-MB-231. At later time points, when the cells have already acquired resistance, MMP-1 expression, secretion and activity are induced upon artesunate treatment. The induction of MMP-1 is well in line with the activation of the tumor progression-associated transcription factors NFκB and AP-1, which are induced upon artesunate treatment as evidenced here by EMSA analyses. It has been reported that both NFκB and AP-1 binding sites are present in the promoter region of MMP-1 [48], [49], [50] and enhanced production of MMP-1 is associated with a more aggressive tumor growth, a higher metastatic potential, and poor clinical outcome of malignant tumors [51], [52], [53].

In conclusion, treatment of MDA-MB-231 cells with artesunate results in development of resistance, which was associated by NFκB and AP-1-mediated apoptosis resistance with up-regulation of the anti-apoptotic *bcl-2* and down-regulations of the pro-apoptotic *bax* genes. The development of resistance towards artesunate may have important implications for the application of this drug in cancer chemotherapy.

## Supporting Information

Table S1 Primer Sequences.
(DOC)
