# Supplementary material for: Development of Resistance towards Artesunate in MDA-MB-231 Human Breast Cancer Cells
Source: PLoS One. 2011 May 26;6(5):e20550. doi: 10.1371/journal.pone.0020550 (PMC3102747; doi:10.1371/journal.pone.0020550)
Supplement: Table S1 — Primer Sequences. (DOC) [file pone.0020550.s001.doc]

**Table S1: Primer Sequences**

| **MMP1** | |  |
| --- | --- | --- |
| sense | 5’-gtgtggtgtctcacagcttcc-3’ | |
| antisense | 5’-aacttgcctcccatcattcttc-3’ | |
| **c-jun** | | |
| sense | 5’-TTACCAAAGGCTAGTGCGATGT-3’ | |
| antisense | 5’-CCACTTGATGCAATCCAAACTT-3’ | |
| **p65** | | |
| sense | 5’-ACGAGCTTGTAGGAAAGGACTG-3’ | |
| antisense | 5’-ATAGGAACTTGGAAGGGGTTGT-3’ | |
| **bcl-2** | | |
| sense | 5’-TGTGGATGACTGAGTACCTG-3’ | |
| antisense | 5’-AGAGACAGCCAGGAGAAATC-3’ | |
| **bax** | | |
| sense | 5’-TCTACTTTGCCAGCAAACT-3’ | |
| antisense | 5’-GGAGGAAGTCCAATGTCCAG-3’ | |
| **bcl-XL** | | |
| sense | 5’-ctcctctcccgacctgtgatac-3’ | |
| antisense | 5’-tccaaagccaagataagattctg-3’ | |
| **bfl-1/A1** | | |
| sense | 5’-gaataacacaggagaatggataagg-3’ | |
| antisense | 5’-tcatccagccagatttaggttc-3’ | |
| **survivin** | | |
| sense | 5’-ACTGAGAACGAGCCAGACTT-3’ | |
| antisense | 5’-CGGACGAATGCTTTTTATGTTC-3’ | |
| **bad** | | |
| sense | 5’-GAGGATGAGTGACGAGTTTGTG-3’ | |
| antisense | 5’-CAAGTTCCGATCCCACCAG-3’ | |
| **RPII** | | |
| sense | 5’-GCACCACGTCCAATGACAT-3’ | |
| antisense | 5’-GTGCGGCTGCTTCCATAA-3’ | |
| **HPRT** | | |
| sense | 5’-CTCAACTTTAACTGGAAAGAATGTC-3’ | |
| antisense | 5’-TCCTTTTCACCAGCAAGCT-3’ | |
| **GAPDH** | | |
| sense | 5’-GAAGGTGAAGGTCGGAGTC -3’ | |
| antisense | 5’-GAAGATGGTGATGGGATTTC-3’ | |
